# YsHyl8A, an Alkalophilic Cold-Adapted Glycosaminoglycan Lyase Cloned from Pathogenic *Yersinia* sp. 298

**DOI:** 10.3390/molecules27092897

**Published:** 2022-05-02

**Authors:** Shilong Zhang, Yujiao Li, Feng Han, Wengong Yu

**Affiliations:** 1 School of Medicine and Pharmacy, Ocean University of China, Qingdao 266003, China; zsl8877@stu.ouc.edu.cn (S.Z.); lyj6476@stu.ouc.edu.cn (Y.L.); 2 Laboratory for Marine Drugs and Bioproducts of Qingdao National Laboratory for Marine Science and Technology, Qingdao 266237, China; 3 Key Laboratory of Marine Drugs, Ministry of Education, Ocean University of China, Qingdao 266003, China; 4 Shandong Provincial Key Laboratory of Glycoscience and Glycoengineering, Ocean University of China, Qingdao 266003, China

**Keywords:** glycosaminoglycan lyase (GAG lyase), chondroitinase, hyaluronidase, cold adaptability, fish pathogens

## Abstract

A high enzyme-yield strain *Yersinia* sp. 298 was screened from marine bacteria harvested from the coastal water. The screening conditions were extensive, utilizing hyaluronic acid (HA)/chondroitin sulfate (CS) as the carbon source. A coding gene *yshyl8A* of the family 8 polysaccharide lyase (PL8) was cloned from the genome of *Yersinia* sp. 298 and subjected to recombinant expression. The specific activity of the recombinase YsHyl8A was 11.19 U/mg, with an optimal reaction temperature of 40 °C and 50% of its specific activity remaining after thermal incubation at 30 °C for 1 h. In addition, its optimal reaction pH was 7.5, and while it was most stable at pH 6.0 in Na_2_HPO_4_-citric acid buffer, it remained highly stable at pH 6.0–11.0. Further, its enzymatic activity was increased five-fold with 0.1 M NaCl. YsHyl8A, as an endo-lyase, can degrade both HA and CS, producing disaccharide end-products. These properties suggested that YsHyl8A possessed both significant alkalophilic and cold-adapted features while being dependent on NaCl, likely resulting from its marine source. *Yersinia* is a typical fish pathogen, with glycosaminoglycan lyase (GAG lyase) as a potential pathogenic factor, exhibiting strong hyaluronidase and chondroitinase activity. Further research on the pathogenic mechanism of GAG lyase may benefit the prevention and treatment of related diseases.

## 1. Introduction

Hyaluronic acid (HA) is a linear macromolecular acid mucopolysaccharide composed of D-glucuronic acid (GlcA) and N-acetylglucosamine (GlcNAc) disaccharide repeating units connected through β-1,3-glycosidic bonds and β-1,4-glycosidic bonds [1,2]. Chondroitin sulfate (CS) is a macromolecular acid mucopolysaccharide in the form of a proteoglycan. CS is widely distributed in a variety of animal tissues, including skin, blood vessels, and vitreous body, and is concentrated in cartilage tissues [3].

Hyaluronidase is used in the treatment of osteoarthritis, to recover from cosmetic surgery, to promote angiogenesis and wound healing, and to regulate inflammation. Chondroitinase can treat arthritis, corneal injury, angina pectoris, and anti-atherosclerosis. Currently, hyaluronidase and chondroitinase are rarely commercially available, so it is necessary to expand their diversity through screening new enzymes [4,5].

While the ocean is a critical source for novel medicinal compounds, it remains less investigated than terrestrial ecosystems, with fewer marine-derived glycosaminoglycan lyases (GAG lyases) reported [6]. Our samples were harvested from the coastal waters of Qingdao and other coastal regions, with marine bacteria collected and screened from rotten fish, squid, and crabs. Bacterial screening utilized HA and CS as the carbon source, resulting in more than 500 strains of marine bacteria that were able to degrade HA and CS. Amongst these strains was *Yersinia* sp. 298, exhibiting the highest enzymatic yield [7]. We cloned a coding gene *yshyl8A* of family 8 polysaccharide lyase (PL8) from the genome of *Yersinia* sp. 298 and subjected it to artificial synthesis and recombinant expression to study its enzymatic properties. In this study, the GAG lyase-producing bacterium, *Yersinia* sp. 298, was screened and identified, with the GAG lyase YsHyl8A purified and characterized.

## 2. Results

### 2.1. Isolation and Identification of Yersinia sp. 298

**Figure 1:**
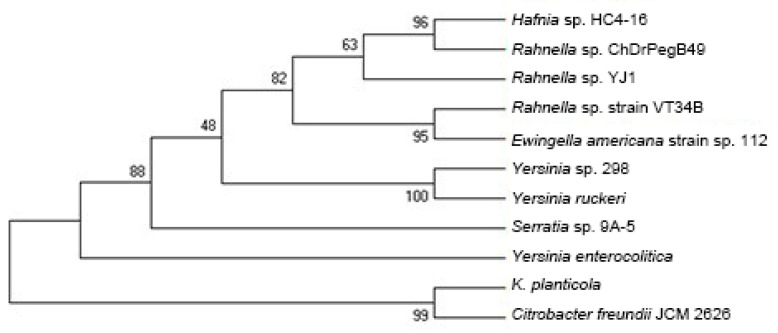
Phylogenetic tree of Strain 298 and related species. The 16S rDNA sequences were used for this analysis. The numbers (0~100) on the branches indicated the reliability of the branch. A larger value means the branch is more reliable.

After detecting the enzymatic activity of the strains producing transparent circles in preliminary screening, 18 strains had higher enzyme-yield activities (>20 U/L), of which strain 298 had the highest enzyme activity (87 U/L). The 16S rDNA sequencing results of strain 298 were over 99% identity to *Yersinia ruckeri*, with strain 298 and *Yersinia ruckeri* branching (Figure 1). Therefore, strain 298 was identified as *Yersinia* genus and named *Yersinia* sp. 298.

Available sequences of *Yersinia* contain sequences of GAG lyase, predicted to belong to PL8. After sequence analysis, we confirmed that the reported sequence was a segment of GAG lyase belonging to PL8. The corresponding forward and reverse primers were designed as follows: F: 5′-GCCATATGCATACACCTTCCAGACTTA-3′ and R: 5′-TGCTCGAGATCGACTGGTTTGACTGTC-3′.

The 16S rDNA and *yshyl8A* gene of strain *Yersinia* sp. 298 have been deposited in GenBank under the accession numbers OM994573 and OM994254, respectively.

### 2.2. Acquisition of Recombinant Sequences

**Figure 2:**
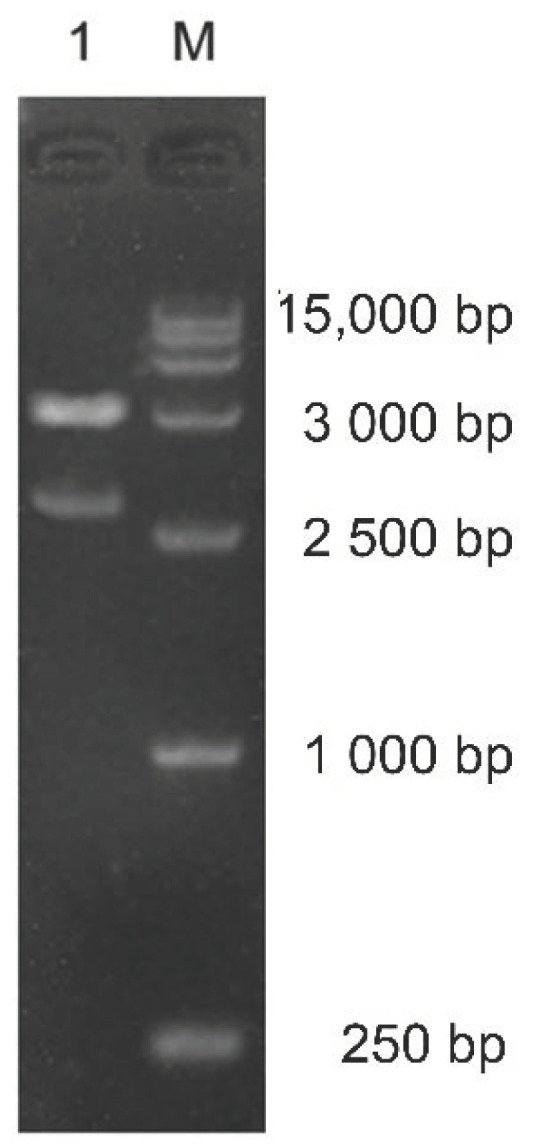
Validation of *yshyl8A* recombinant sequences: 1, Digestion validation results; M, 15,000 DNA Marker.

The full-length sequence of *yshyl8A* was 3114 bp. It could encode a polypeptide of 1037 amino acid residues. The theoretical molecular weight of YsHyl8A was 115.4 kDa, and its theoretical isoelectric point (*pI*) value was 6.7. PCR using the designed recombinant primers with the genome of *Yersinia* sp. 298 as a template produced a sequence of approximately 3000 bp in length. This sequence was ligated into the pMD18-simple T vector and transformed into *Escherichia coli* DH5α, and the plasmid was extracted. The positive clones were selected for sequencing, and the plasmids were extracted from clones with correct sequences and digested with two restriction enzymes (*Nde* I and *Xho* I). A fragment, approximately 3000 bp, was used for gel extraction, and its concentration was determined for later use (Figure 2).

### 2.3. Expression and Purification of YsHyl8A

**Figure 3:**
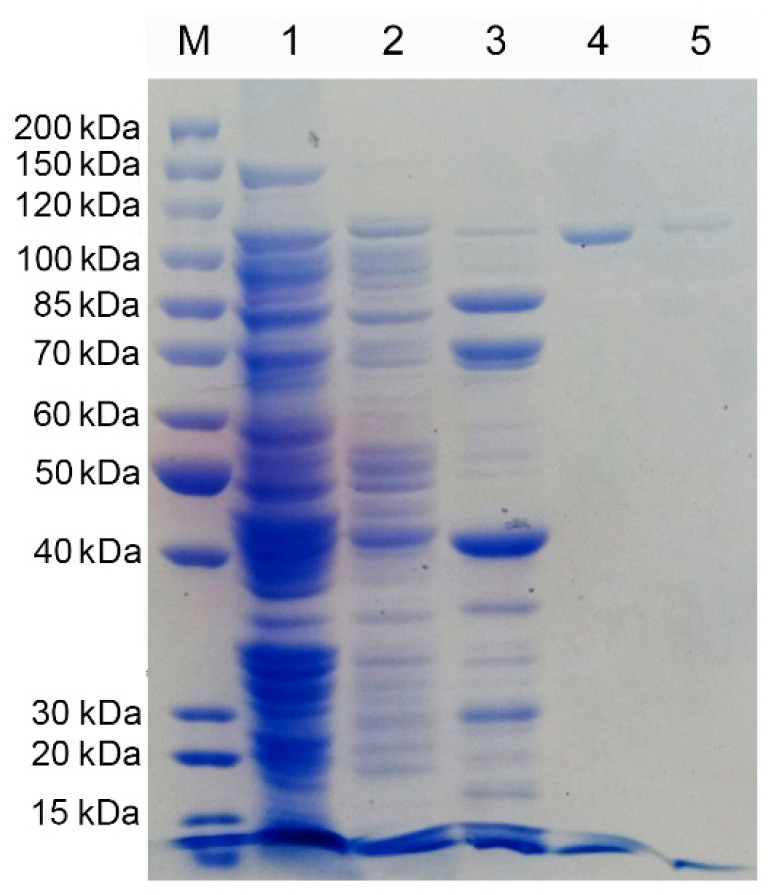
SDS-PAGE of purification of recombination YsHyl8A: M, Protein Marker; 1, Breakthrough peak; 2, 25 mM imidazole elution; 3, 100 mM imidazole elution; 4, 200 mM imidazole elution; 5, 300 mM imidazole elution.

The *yshly8A* gene was over-expressed in the pET-28a (+)/*E. coli* BL21 (DE3) system. Most of the recombinant proteins existed in the soluble state when the cells were induced at 20 °C for 12 h. The recombinant YsHyl8A protein was purified to homogeneity with a final yield of 22% using Ni-Sepharose column chromatography (Figure 3).

### 2.4. Substrate Specificity of YsHyl8A

**Figure 4:**
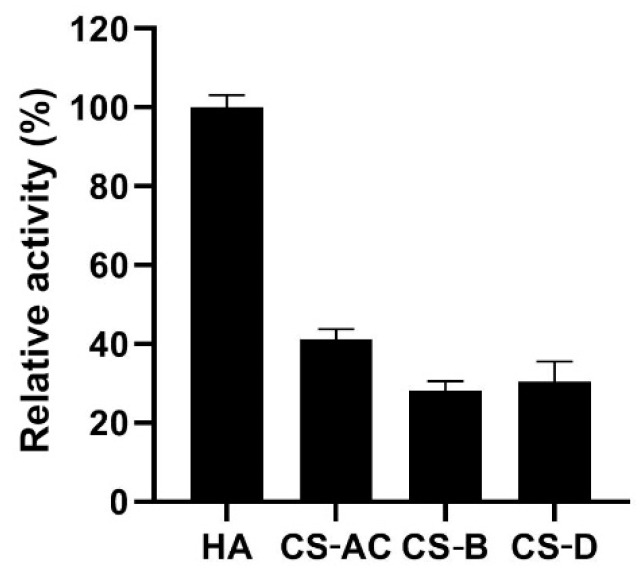
Substrate specificity of YsHyl8A. The activity of YsHyl8A towards HA was defined as 100%. Error bars indicate the standard deviation (*n* = 3).

The degradation substrate specificity of YsHyl8A was characterized through the detection of the enzyme activity using different substrates, with the value of YsHyl8A using HA as 100% (Figure 4).

GAG lyase YsHyl8A had a broad spectrum in its degradation ability, and it was able to degrade various substrates such as HA, CS-AC, CS-B, and CS-D.

### 2.5. Optimal Reaction Temperature and Temperature Stability of YsHyl8A

**Figure 5:**
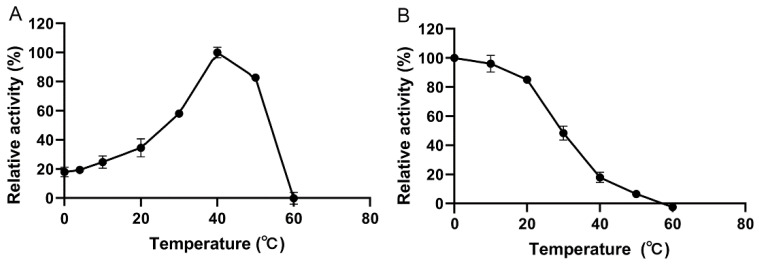
Optimal reaction temperature and temperature stability of YsHyl8A: (**A**) Optimal reaction temperature; (**B**) The temperature stability. The initial activity was defined as 100%. Error bars indicate the standard deviation (*n* = 3).

The enzymatic activity of the separated and purified YsHyl8A was detected at different temperatures, based on which the optimal reaction temperature was determined (Figure 5A).

The optimal reaction temperature of YsHyl8A was 40 °C, while its activity remained high at 0–30 °C, suggesting that it has good cold adaptability. Its enzymatic reaction capacity was almost lost at 60 °C.

The temperature stability of YsHyl8A was characterized through the detection of its residual activity after standing at different temperatures for 1 h. More than 80% of its activity remained after pre-incubation at 0–20 °C, while only 50% remained at 30 °C (Figure 5B). With further temperature increases, almost all enzyme activity was lost. YsHyl8A showed limited tolerance to increasing temperatures, again likely related to its original marine source, and the enzymatic reaction was normally conducted at lower temperatures.

### 2.6. Optimal Reaction pH and pH Stability of YsHyl8A

**Figure 6:**
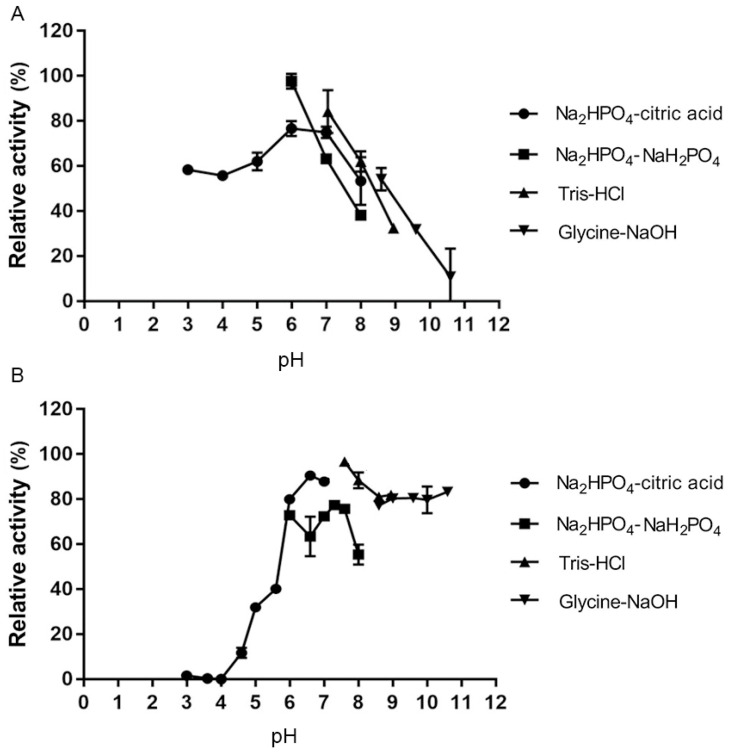
The optimal pH and pH stability of YsHyl8A: (**A**) Optimal pH; (**B**) pH stability. HA was used as the substrate. The activity of YsHyl8A at the optimal pH and temperature was defined as 100%. Error bars indicate the standard deviation (*n* = 3).

The optimal reaction pH was determined in four different pH buffer systems. The enzyme activity of YsHyl8A was higher in Na_2_HPO_4_-NaH_2_PO_4_ buffer and peaked at pH 6.0 (Figure 6A). The catalytic activity of YsHyl8A also remained high at pH 3–8.5.

The pH stability of YsHyl8A was characterized through the detection of its residual activity after standing in different pH buffer systems for 6 h (Figure 6B).

YsHyl8A was stable at pH 6.0–11.0, with 80% of activity remaining. It was most stable at pH 7.5 in Tris-HCl buffer, indicating its highly alkalophilic nature.

### 2.7. Effects of SDS, EDTA, and Different Metal Ions on the Activity of YsHyl8A

**Figure 7:**
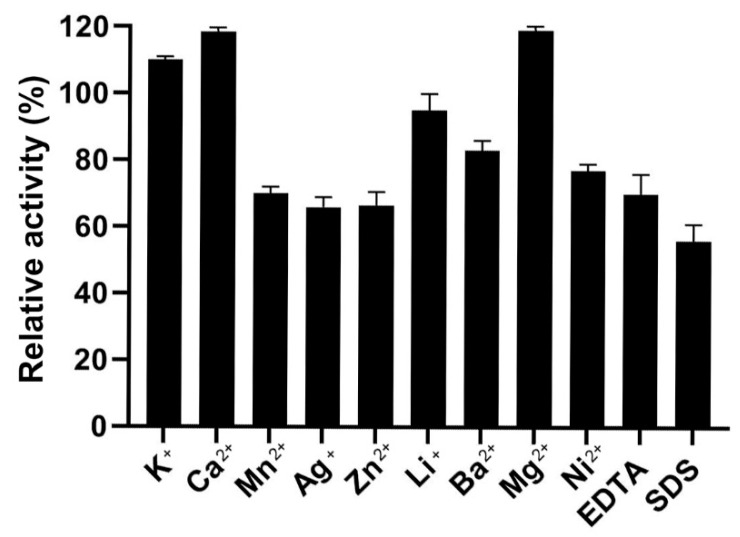
Effect of reagents on enzyme activity of YsHyl8A. Effects of metal ions, chelator (EDTA, 1 mM), and surfactant (SDS, 0.1%, *w*/*v*). Error bars indicate the standard deviation (*n* = 3).

The effects of different ions and detergents on the activity of YsHyl8A were characterized by detecting the activity of YsHyl8A against HA in the presence of different metal ions, EDTA, and SDS, respectively (Figure 7).

Ca^2+^ and Mg^2+^ enhanced the activity of YsHyl8A, while Mn^2+^, Ag^+^, Zn^2+^, Ba^2+^, and Ni^2+^ inhibited its activity to varying degrees, and K^+^ had little influence (Figure 7). Both EDTA and SDS suppressed the activity of YsHyl8A, suggesting that, not only would the activity of YsHyl8A be affected if the metal ions in the system are chelated, but also the denaturant may have a certain effect on the activity.

### 2.8. Effects of NaCl on the Activity of YsHyl8A

**Figure 8:**
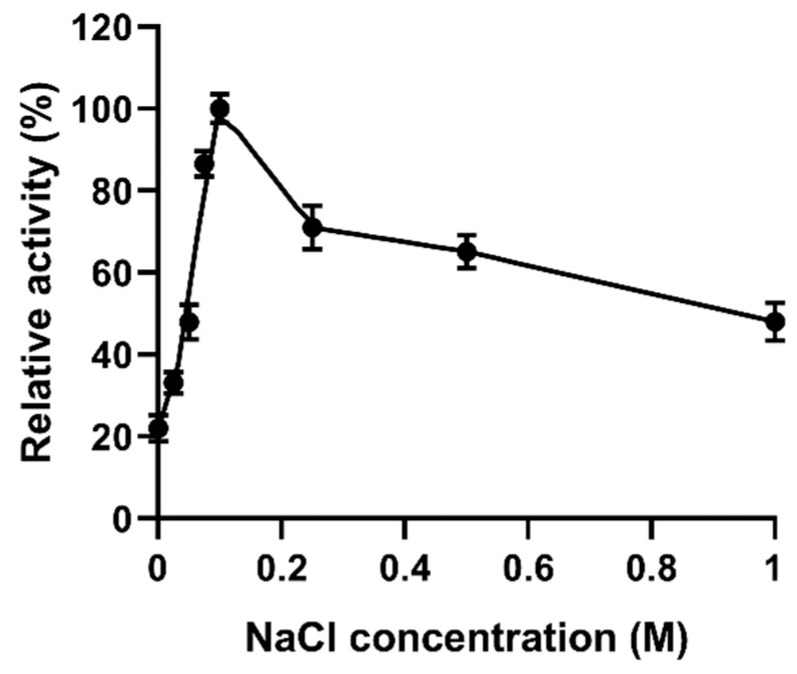
Effect of NaCl on enzyme activity of YsHyl8A. The activity of YsHyl8A at the optimal NaCl concentration was defined as 100%. Error bars indicate the standard deviation (*n* = 3).

YsHyl8A maintained a certain activity in the absence of NaCl, and the addition of 0.1 mM NaCl could increase its activity by five times (Figure 8). With the further increase in NaCl concentration, the activity of YsHyl8A declined, which may be related to its marine origin.

### 2.9. Mode of Enzymatic Degradation

**Figure 9:**
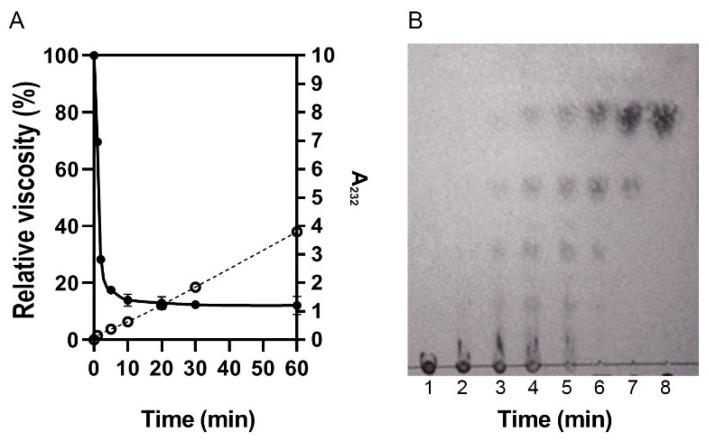
Viscosity analysis of enzymatic pattern and TLC analysis of the reaction pattern of enzyme: (**A**) Viscosity analysis; (**B**) TLC analysis, from left to right are 0, 5, 10, 20, 30, 60, 120, 240 min, respectively.

The mode of action of YsHyl8A was detected by using the viscosity method and TLC analysis. Under random endo-digestion, the GAG endo-lyase first degrades macromolecular GAG into smaller fragments, dramatically changing the viscosity of the reaction system in the early stage of enzymatic degradation, once a small concentration of endo-lyase is added. In contrast, the exo-lyase cleaves oligosaccharides one by one from a segment of macromolecular GAG, slowly changing the viscosity. Therefore, the mode of action of the enzymes (endo- or exo-digestion) can be intuitively determined based on the change in enzymatic activity combined with the corresponding change in viscosity. In this experiment, 1 U/mL YsHyl8A was added into the reaction system, and the viscosity changes at different degradation times were detected using a viscometer (Figure 9A).

GAG lyases can be classified into endo-lyases and exo-lyases according to different modes of degradation. The degradation end-products of endo-lyases are generally disaccharides and tetrasaccharides, while those of exo-lyases are disaccharides [8,9]. Moreover, a large number of oligosaccharide units in the intermediate fragment emerge during enzymatic degradation by endo-lyases [10]. With the continuous degradation, the oligosaccharides in the intermediate fragment gradually become the end-products. On the contrary, the exo-lyases gradually degrade the polysaccharide fragment from one end, so that no oligosaccharides in the intermediate fragment emerge during enzymatic degradation.

Therefore, the degradation mode of YsHyl8A was detected by analyzing the products of degradation at different times (Figure 9).

The viscosity of GAG produced at different times was measured using the Ubbelohde viscometer (Figure 9A). During the early stages of the enzymatic reaction, the activity of YsHyl8A was lower, while the viscosity sharply declined, preliminarily confirming the degradation mode to be endo-digestion. The results of the TLC revealed that the initial products of YsHyl8A in the enzymatic degradation were mainly the oligosaccharides in the intermediate fragment, further confirming the degradation mode to be endo-digestion (Figure 9B).

### 2.10. MS Identification of Degradation End-Products

**Figure 10:**
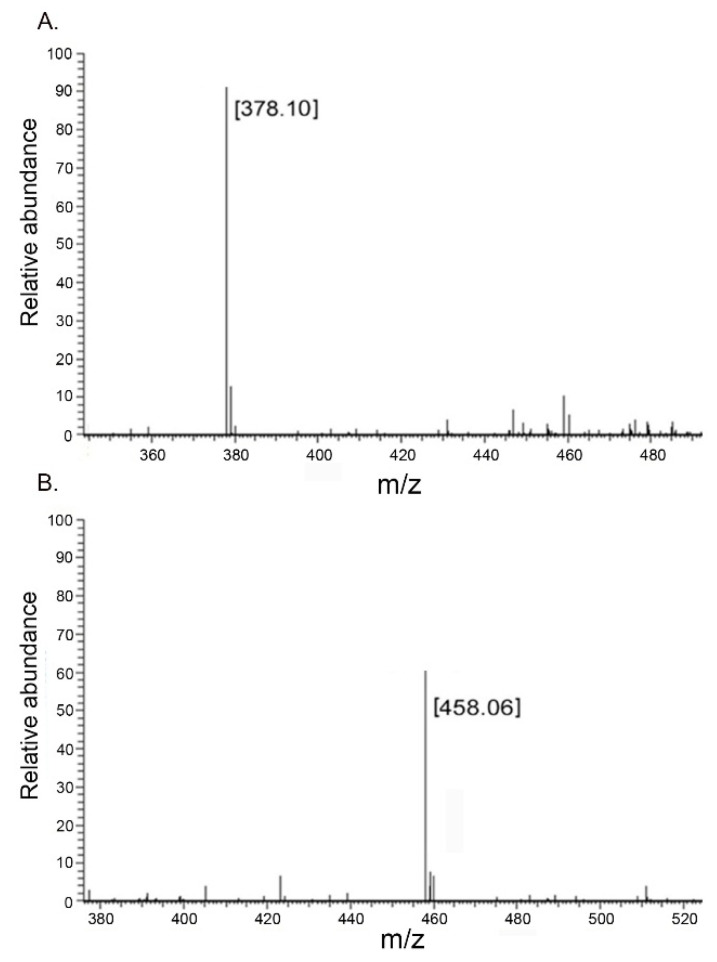
ESI-MS spectrum of purified degradation end-products of YsHyl8A: (**A**) The degradation result of YsHyl8A towards HA; (**B**) The degradation result of YsHyl8A towards CS.

The degradation end-products were harvested and subjected to ESI-MS. As the result, the two major signals were 378.11 m/z and 458.06 m/z (Figure 10), consistent with the molecular mass of the non-sulfated unsaturated disaccharide Δ^4,5^ HexUA-GlcNAc and the monosulfated unsaturated disaccharide Δ^4,5^ HexUA-GalNAc (4S), respectively. This demonstrated that YsHyl8A was an endo-lyase whose degradation end-products were disaccharides.

## 3. Discussion

The coding gene, *yshyl8A* of GAG lyase, was cloned from *Yersinia* sp. 298. It contained 3114 base sequences that could encode a polypeptide of 1037 amino acid residues, whose theoretical molecular weight was 115.4 kDa and theoretical *pI* value was 6.7. It also contained a signal peptide composed of 27 AA at the N-terminus, and its amino acid residue sequence was 99% identity to the deduced chondroitin lyase of the *Yersinia* genus in the CAZy database. This similar sequence in the *Y. ruckeri* genome was predicted to be chondroitin lyase, but there was no report on its properties. *Y. ruckeri* is a pathogenic bacterium of silver carp and bighead carp, and GAG lyase may be one of its pathogenic factors. YsHyl8A is potentially a pathogenic factor of the *Yersinia* genus, therefore increasing its importance for further analysis. With expression vectors constructed using pET-28a (+), GAG lyase YsHyl8A was subjected to efficient expression in *E. coli*. Additionally, the optimal enzyme-producing conditions were preliminarily determined by exploring the inducer concentration and induction time: the enzyme yield reached 150 U/L after induction with 0.1 mM IPTG at 20 °C and 160 rpm for 24 h [11].

After efficient expression strains were constructed, the recombinant enzyme was separated and purified through the Ni-affinity column (HisTrap HP). After one-step purification, the recombinant enzyme was electrophoretically pure, and the protein recovery reached 22%.

YsHyl8A was a bacterial protein isolated from marine fish, and its enzymatic properties also exhibited dependence on NaCl and a preference for low-temperature environment. Its specific activity was 11.19 U/mg, and its optimal reaction temperature was 40 °C, below which the activity remained high. YsHyl8A showed high relative catalytic activity at 0–30 °C; this property is also excellent in the reported cold-adapted enzymes [12]. However, its catalytic capacity was almost lost at 60 °C. Only 50% of its specific activity remained after thermal insulation at 30 °C for 1 h. The enzymatic activity was stable at 0–20 °C, and with further temperature increases, almost all enzyme activity was lost. We posit that YsHyl8A is a cold-adapted enzyme exhibiting poor tolerance to increased temperatures. Its optimal reaction pH was 7.5, and it was most stable at pH 6.0 in Na_2_HPO_4_-citric acid buffer. The catalytic activity of YsHyl8A was stable at pH 6–11, with 80% of activity remaining. This property of maintaining high activity over a wide range of alkaline pH is rare among reported glycosaminoglycan lyases. EDTA and SDS could inhibit its enzyme activity. YsHyl8A maintained a certain activity in the absence of NaCl, and 100 mM NaCl could increase its activity several times. With the further increase in NaCl concentration, the enzyme activity declined. This property potentially results from its marine origin. In addition, we studied the mode of degradation and identified the degradation end-products. Finally, we determined that YsHyl8A is an endo-lyase whose degradation end-products are disaccharides HA and CS [13,14,15].

## 4. Materials and Methods

### 4.1. Isolation and Identification of Bacteria

Bacterial plates were incubated in a unique carbon source screening medium at 25 °C for 3 days to allow for the formation of the detectable colonies. They were identified by pouring 10 mL of 200 mM glacial acetic acid onto the surface of the plate and observing whether transparent circles were formed. At least 500 strains were inoculated into a selective medium without agar and assayed for GAG lyase activity in the culture supernatant. Bacterial growth was evaluated by measuring absorbance at 600 nm [16]. The *16S rRNA* gene of each strain was amplified with PCR from the extracted genomic DNA and sequenced. The obtained *16S rRNA* gene sequence was blasted and aligned with its closely related sequences retrieved from GenBank using the BLASTn and CLUSTAL X.

### 4.2. Enzyme Activity of YsHyl8A

The concentration of products generated by light absorption of unsaturated double bonds in the reaction system was quantitatively detected by absorbance at 232 nm (A_232_). The reaction between GAG lyase and GAG substrate produces unsaturated double bonds with a maximum A_232_, and their abundance is directly proportional to the absorbance value within a certain range. Therefore, the concentration of unsaturated double bonds in the substrate can be detected by measuring the A_232_, with each enzyme activity determined by displaying the abundance of final products [17].

The samples (20 µg enzyme in 100 µL) were added to 900 µL of GAG substrate (0.2%, *w*/*v*) dissolved in 10 mM Tris-HCl buffer (pH 7.5), mixed well, subjected to a thermostatic water bath at 37 °C for 10 min, boiled for 10 min, and cooled to room temperature. Their absorbance at A_232_ was then measured, with the enzyme solution inactivated by boiling as a control. The enzyme concentration required to catalyze the production of 1 μmol of reducing sugar per minute was defined as one unit (U) of enzymatic activity [18].

### 4.3. YsHyl8A Sequence Analysis

The full-length sequence of YsHyl8A was spliced, and primers were designed for sequence verification to determine its DNA sequence. The signal peptide was predicted using the prediction software SignalP 4.0 Server (available online: http://www.cbs.dtu.dk/services/SignalP/ (accessed on 10 January 2019)); pI/Mw was predicted with the Compute pI/Mw tool (available online: http://web.expasy.org/computepi/ (accessed on 10 January 2019)); and the similarity in sequences between YsHyl8A and other proteins was predicted using BLAST. The restriction site was predicted with the Primer 5 software.

### 4.4. Acquisition of Recombinant Gene, and Expression and Purification of Recombinant YsHyl8A

The primers possessed *Nde* I and *Xho* I sites at their 5′ ends. Primers: F-primer (5′-GCCATATGCATACACCTTCCAGACTTA-3′) and R-primer (5′-TGCTCGAGATCGACTGGTTTGACTGTC-3′).

The PCR product was digested with *Nde* I and *Xho* I and ligated into the pET-28a (+) vector previously digested with *Nde* I and *Xho* I. The recombinant plasmid, pET-28a-YsHyl8A, was transferred into *E. coli* BL21 (DE3). Protein expression was induced at OD_600_ of 0.6 with 0.05 mM isopropyl-*β*-thiogalactoside (IPTG) for 12 h at 20 °C and 120 rpm [19]. The bacterial cells were harvested and sonicated in the lysis buffer (20 mM phosphate buffer (pH 7.5), 500 mM NaCl, 10 mM imidazole). The soluble fraction of protein was obtained by centrifugation at 10,000× *g* at 4 °C for 30 min, and then loaded on the Ni-Sepharose column. The column was washed with the wash buffer (20 mM phosphate buffer (pH 7.5), 500 mM NaCl, 10 mM imidazole), and the target protein was eluted with elution buffer (20 mM phosphate buffer (pH 7.5), 500 mM NaCl, 200 mM imidazole). The elution fraction was desalted by dialysis with 50 mM Tris-HCl buffer (pH 7.5) as the mobile phase. The Mw of YsHyl8A was determined with sodium dodecyl sulfate polyacrylamide gel electrophoresis (SDS-PAGE) [20,21]. The protein concentration was determined using the method of Lowry and colleagues using bovine serum albumin (BSA) as the standard [22].

### 4.5. Detection of Substrate Specificity

Chondroitin AC, B, and HA were purchased from Sigma. Dermatan sulfate was purchased from Huasheng Group (Qingdao, China) (purity: ~99%) [8]. All other chemicals and reagents were of the highest quality available. *E. coli* strains DH5α and BL21 (DE3) were grown at 37 °C in Luria–Bertani (LB) broth supplemented with either ampicillin (50 µg/mL) or kanamycin (30 µg/mL) if necessary [23].

The degradation preference of YsHyl8A to different substrates was determined using several different substrates (HA, CS-B, CS-AC, and CS-D). Specifically, the electrophoretically pure GAG lyase YsHyl8A, obtained by separation and purification, was diluted to an appropriate concentration and reacted with 0.2% HA, CS-B, CS-AC, and CS-D, respectively. Later, its degradation activity for different substrates was measured by A_232_, to compare its degradation preference to other substrates, with its degradation activity for HA as 100% [9,24].

### 4.6. Optimal Reaction Temperature

The activity of YsHyl8A was determined at a range of reaction temperatures, including 0, 4, 10, 20, 30, 40, 50, and 60 °C. After thermal insulation for 10 min, 0.2% (*w*/*v*) HA substrates were added to GAG lyase YsHyl8A, and the degradation activity of YsHyl8A was detected, with the value measured at the reaction temperature when the enzymatic activity was the highest as 100% [25].

### 4.7. Temperature Stability

The temperature stability of YsHyl8A was characterized by detecting the enzymatic activity after standing at different temperatures for 1 h. Specifically, after YsHyl8A was placed at 0, 10, 20, 30, 40, 50, and 60 °C for 1 h and then quickly cooled to 0 °C, its activity was detected at the optimal reaction temperature, with the enzymatic activity at the optimal reaction temperature before incubation as 100% [23,26].

### 4.8. Optimal Reaction pH and pH Stability

The optimal reaction pH of YsHyl8A was identified by detecting its activity at different pH values. Specifically, 50 mM substrates (pH 3.0–11.0) containing 0.2% (*w*/*v*) HA were prepared using four different reaction systems: Na_2_HPO_4_-citric acid buffer (pH 3.0–7.0), Tris-HCl buffer (pH 7.0–9.0), Na_2_HPO_4_-NaH_2_PO_4_ buffer (pH 6.0–8.0), and glycine-NaOH buffer (pH 8.6–11.0). The highest enzymatic activity was defined as 100%, under which the pH was the optimal reaction pH.

The pH stability of YsHyl8A was similarly characterized after standing in these four reaction systems for 6 h, where the enzymatic activity before incubation was defined as 100% [26].

### 4.9. Effect of NaCl on the Activity of YsHyl8A

The activity of YsHyl8A was determined after the final concentration of NaCl in the substrate was adjusted to 0 M, 0.025 M, 0.05 M, 0.075 M, 0.1 M, 0.25 M, 0.5 M, and 1 M, respectively [27].

### 4.10. Effects of Metal Ions and Inhibitors on the Activity of YsHyl8A

The effects of metal ions on the activity of purified YsHyl8A were examined by using various metal ions (1 mM), chelators (EDTA, 1 mM), and surfactant (SDS, 0.1%, *w*/*v*) [28,29].

### 4.11. Analysis of Degradation Pattern and Degradation Products of YsHyl8A

With 0.1% (*w*/*v*) HA as the substrate, the degradation mode of YsHyl8A was identified by determining the viscosity change and degradation products during degradation. Specifically, 10 U of YsHyl8A was added to 15 mL of 0.1% HA substrate for enzymatic degradation for 1, 2, 5, 10, 20, 30, and 60 min, respectively. Then, the degradation mode of YsHyl8A was analyzed based on the viscosity change in the substrate after degradation (viscosity method) and the degradation product analysis (thin layer chromatography, TLC). TLC: 2 μL of products of degradation for different times were spotted onto the TLC plate, followed by plate development with the developing agent (n-butanol/glacial acetic acid/water = 2/1/1; *v*/*v*/*v*) and 5-min baking at 130 °C for color development with aniline diphenylamine [29,30].

### 4.12. Analysis of Degradation End-Products of YsHyl8AG

YsHyl8A (200 U) was added to 100 mL of 0.2% HA and CS for thorough degradation at 30 °C for 12 h. Fast protein liquid chromatography was performed on the degradation end-products, and the separated peaks were collected for electrospray ionization mass spectrometry [31].

## 5. Conclusions

In this paper, we recombinantly expressed and subsequently purified a novel glycosaminoglycan lyase, named YsHyl8A. According to the multiple enzymatic properties of YsHyl8A, it is a protein highly in line with the strain growth environment, namely, the high-salt and cold-adaptive environment. Further research on its enzymatic properties and protein structure–function relationship will help to elucidate the role of GAG lyase in the pathogenic process of pathogenic bacteria and the necessary conditions for its function, thereby contributing to the targeted development of marine fish drugs. Through investigations into the key protein structure that determines its cold-adapted properties, and comparison to the key structures of other heat-resistant proteins, the directional transformation into thermophilic and cold-adapted proteins will be strongly benefited.

## Data Availability

Not available.
